# The CCL2/CCR2 Axis Enhances Vascular Cell Adhesion Molecule-1 Expression in Human Synovial Fibroblasts

**DOI:** 10.1371/journal.pone.0049999

**Published:** 2012-11-21

**Authors:** Yu-Min Lin, Chin-Jung Hsu, Yuan-Ya Liao, Ming-Chih Chou, Chih-Hsin Tang

**Affiliations:** 1 Institute of Medicine, Chung Shan Medical University, Taichung, Taiwan; 2 Department of Orthopedic Surgery, Taichung Veterans General Hospital, Taichung, Taiwan; 3 School of Chinese Medicine, China Medical University, Taichung, Taiwan; 4 Department of Orthopaedics, China Medical University Hospital, Taichung, Taiwan; 5 Department of Surgery, Chung Shan Medical University Hospital, Taichung, Taiwan; 6 Department of Pharmacology, School of Medicine, China Medical University, Taichung, Taiwan; 7 Graduate Institute of Basic Medical Science, China Medical University, Taichung, Taiwan; University Hospital Freiburg, Germany

## Abstract

**Background:**

Chemokine ligand 2 (CCL2), also known as monocyte chemoattractant protein-1 (MCP-1), belongs to the CC chemokine family that is associated with the disease status and outcomes of osteoarthritis (OA). Here, we investigated the intracellular signaling pathways involved in CCL2-induced vascular cell adhesion molecule-1 (VCAM-1) expression in human OA synovial fibroblasts (OASFs).

**Methodology/Principal Findings:**

Stimulation of OASFs with CCL2 induced VCAM-1 expression. CCL2-mediated VCAM-1 expression was attenuated by CCR2 inhibitor (RS102895), PKCδ inhibitor (rottlerin), p38MAPK inhibitor (SB203580), and AP-1 inhibitors (curcumin and tanshinone IIA). Stimulation of cells with CCL2 increased PKCδ and p38MAPK activation. Treatment of OASFs with CCL2 also increased the c-Jun phosphorylation and c-Jun binding to the AP-1 element on the VCAM-1 promoter. Moreover, CCL2-mediated CCR2, PKCδ, p38MAPK, and AP-1 pathway promoted the adhesion of monocytes to the OASFs monolayer.

**Conclusions/Significance:**

Our results suggest that CCL2 increases VCAM-1 expression in human OASFs via the CCR2, PKCδ, p38MAPK, c-Jun, and AP-1 signaling pathway. The CCL2-induced VCAM-1 expression promoted monocytes adhesion to human OASFs.

## Introduction

Osteoarthritis (OA) is a chronic joint disorder characterized by slow progressive degeneration of articular cartilage, subchondral bone alteration, and variable secondary synovial inflammation. In response to macrophage-derived proinflammatory cytokines such as interleukin (IL)-1β and tumor necrosis factor-α (TNF-α), OA synovial fibroblasts (OASFs) produce chemokines that promote inflammation, neovascularization, and cartilage degradation via activation of matrix-degrading enzymes such as matrix metalloproteinases (MMPs) [1], [2]. Although the pathogenesis of the disease remains elusive, there is increasing evidence indicating that mononuclear cells migration plays an important role in the perpetuation of inflammation in synovium [3], [4]. Adhesion and infiltration of mononuclear cells to inflammatory sites are regulated by adhesion molecules, such as vascular adhesion molecule-1 (VCAM-1) [5], [6].

Cell adhesion molecules are transmembranes glycoprotein that mediates cell-cell and cell-extracellular matrix interactions. VCAM-1 has recently emerged as a highly significant predictor of the risk of OA [7], [8]. Up-regulation of VCAM-1 has been shown in the synovial lining of OA patients by immunohistochemical staining and in cultured human OASFs by Western blotting [7], [8]. Reducing the levels of VCAM-1 in synovial fluid may suppress the inflammatory response in knee OA [9]. VCAM-1 is involved in the process of infiltration of synovium with mononuclear cells leading to the initiation and progression of the disease. However, the molecular mechanisms by which cytokines induce VCAM-1 expression in human OASFs remain unclear.

Chemokines are low molecular weight secretory proteins that can regulate the chemotaxis and metabolic activity of specific leukocyte subsets. Monocyte chemoattractant protein 1 (MCP-1)/chemokine ligand 2 (CCL2), a ligand of CCR2, is chemotactic for monocyte/macrophages and activated T cells [10], [11]. It was reported that the levels of CCL2 are increased in the blood, synovial fluid, and synovial tissue of patients with OA and rheumatoid arthritis (RA) [12], [13]. Injection of CCL2 into rabbit joints resulted in marked macrophage infiltration in the affected joint [14]. Treatment with CCL2 antagonist before disease onset in an MRL/lpr mouse model of arthritis was shown to prevent the onset of arthritis [15]. These data suggest that CCL2 plays an important role during OA pathogenesis.

Although the roles of cytokines and adhesion molecules in polymorphonuclear cells adhesion to endothelial cells have been described in detail, little is known about the mechanisms underlying the interaction between monocytes and human OASFs. Previous studies have shown that CCL2 plays important role in OA pathogenesis [16], [17]. In the present study, we explored the possible intracellular signaling pathways involved in CCL2-induced VCAM-1 expression in human OASFs. The results show that CCL2 activates the CCR2 receptor which in turn activates protein kinase Cδ (PKCδ), p38MAPK, and AP-1 signaling pathway, leading to the upregulation of VCAM-1 expression. The increased VCAM-1 expression correlates with enhanced adhesion of monocytes to CCL2-stimulated OASFs.

## Materials and Methods

### Materials

Protein A/G beads; anti-mouse and anti-rabbit IgG-conjugated horseradish peroxidase; rabbit polyclonal antibodies specific for PKCδ, p38MAPK, p-p38MAPK(Tyr182) (sc-7973), c-Jun, p-c-Jun(Ser73) (sc-16311-R), and β-actin; and siRNA against PKCδ and c-Jun were purchased from Santa Cruz Biotechnology (Santa Cruz, CA, USA). Rabbit polyclonal antibody specific for PKCδ phosphorylated at Tyr^331^ was purchased from Cell Signaling and Neuroscience (Danvers, MA, USA). Rottlerin, GF109203X, SB203580, curcumin, and tanshinone IIA were purchased from Calbiochem (San Diego, CA, USA). Recombinant human CCL2 was purchased from R&D Systems (Minneapolis, MN, USA). The p38MAPK dominant negative mutant was provided by Dr. J. Han (University of Texas South-western Medical Center, Dallas, TX). All other chemicals were obtained from Sigma-Aldrich (St. Louis, MO, USA).

### Cell Cultures

The study protocol was approved by the Institutional Review Board of China Medical University Hospital, and all subjects gave informed written consent before enrollment. Human synovial fibroblasts were isolated using collagenase treatment of synovial tissues obtained from knee replacement surgeries of 33 patients with OA and 15 samples of normal synovial tissues obtained at arthroscopy from trauma/joint derangement. The synovial fluid concentration of CCL2 was measured with an enzyme-linked immunosorbent assay (ELISA) according to the protocol provided by the manufacturer (Human CCL2 ELISA kit; R&D systems, Minneapolis, MN). OASFs were isolated, cultured, and characterized as previously described [18], [19]. Experiments were performed using cells from passages 3 to 6.

THP-1, a human leukemia cell line of monocyte/macrophage lineage, was obtained from American Type Culture Collection (Manassas, VA, USA) and grown in RPMI-1640 medium with 10% fetal bovine serum.

### Quantitative Real-time PCR

Total RNA was extracted from OASFs using a TRIzol kit (MDBio Inc., Taipei, Taiwan). The reverse transcription reaction was performed using 2 µg of total RNA that was reverse transcribed into cDNA using oligo (dT) primer [20], [21]. The quantitative real-time PCR (qPCR) analysis was carried out using Taqman® one-step PCR Master Mix (Applied Biosystems, Foster City, CA). cDNA templates (2 µl) were added per 25-µl reaction with sequence-specific primers and Taqman® probes. Sequences for all target gene primers and probes were purchased commercially (β-actin was used as internal control) (Applied Biosystems). The qPCR assays were carried out in triplicate on an StepOnePlus sequence detection system. The cycling conditions involved 10-min polymerase activation at 95°C, followed by 40 cycles at 95°C for 15 s and 60°C for 60 s. The threshold was set above the non-template control background and within the linear phase of the target gene amplification to calculate the cycle number at which the transcript was detected (denoted CT). Reactions were normalized to copies of β-actin mRNA within the same sample using the −ΔΔCT method. The levels of mRNA are expressed as the fold change in expression level compared with that of controls.

### Western Blot Analysis

Cellular lysates were prepared as described previously [22], [23]. Proteins (30 µg) were resolved on SDS-PAGE and transferred to immobilon polyvinyldifluoride (PVDF) membranes. The blots were blocked with 4% BSA for 1 h at room temperature and then probed with rabbit anti-human antibodies against PKCδ, VCAM-1, p38MAPK, or p-p38MAPK (β-actin was used as loading control) (1∶1000) for 1 h at room temperature. After three washes, the blots were subsequently incubated with donkey anti-rabbit peroxidase-conjugated secondary antibody (1∶1000) for 1 h at room temperature. The blots were visualized by enhanced chemiluminescence with Kodak X-OMAT LS film (Eastman Kodak, Rochester, NY).

### Flow Cytometry Analysis

Human synovial fibroblasts were plated in six-well dishes. The cells were then washed with PBS and detached with trypsin at 37°C. Cells were fixed for 10 min in PBS containing 1% paraformaldehyde. After being rinsed in PBS, the cells were incubated with mouse anti-human antibody against VCAM-1 (1∶100) for 1 h at 4°C. Cells were then washed again and incubated with fluorescein isothiocyanate-conjugated goat anti-mouse secondary IgG (1∶100; Leinco Technologies Inc., St. Louis, MO, USA) for 45 min (Isogenic control antibody was used to detect the background fluorescence) and analyzed by flow cytometry using FACS Calibur (10000 cells were collected for each experiment) and CellQuest software (BD Biosciences).

### Transfection of siRNAs

ON-TARGETplus siRNA of PKCδ, c-Jun, and control were purchased from Dharmacon Research (Lafayette, CO). Transient transfection of siRNAs was carried out using Lipofectamine 2000 transfection reagent. siRNA (100 nM) was formulated with Lipofectamine 2000 transfection reagent according to the manufacturer's instruction.

### Cell Adhesion Assay

THP-1 cells were labeled with BCECF-AM (10 µM) at 37°C for 1 h in RPMI-1640 medium and subsequently washed by centrifugation. OASFs grown on glass coverslips were incubated with CCL2 for 6 h. Confluent CCL2-treated OASFs were incubated with THP-1 cells (2×10^6^ cells/ml) at 37°C for 1 h. Non-adherent THP-1 cells were then removed and gently washed with PBS. The number of adherent THP-1 cells was counted in four randomly chosen fields per well at 200X high power using a fluorescence microscope (Zeiss, Axiovert 200 M).

### Chromatin Immunoprecipitation Assay

Chromatin immunoprecipitation analysis was performed as described previously [24]. DNA immunoprecipitated by anti-c-Jun antibody was purified. The DNA was then extracted with phenol-chloroform. The purified DNA pellet was subjected to PCR, and PCR products were resolved using 1.5% agarose gel electrophoresis and visualized by UV light. The primer 5′-CGGTTAAATCTCACAGCCCA-3′ and the reverse primer 5′-TTCTCTTACAAGAGAAAGGA-3′ (−403 to −30; contain AP-1 binding site). The forward primer 5′-CCAATGGGGGAGATAGACCT-3′) and the reverse primer 5′-ACCGCAAACCCAGTTAAAAA-3′ (−1015 to −775; dose not contain AP-1 binding site) (MDBio Inc., Taipei, Taiwan) were specifically designed to correspond to the VCAM-1 promoter region [24].

### Statistics

The values reported are means ± S.E. Statistical comparisons between two samples were performed using Student’s *t-*test. Statistical comparisons of more than two groups were performed using one-way analysis of variance (ANOVA) with Bonferroni’s *post-hoc* test. In all cases, *p*<0.05 was considered significant.

## Results

### CCL2 Induces VCAM-1 Expression in Human Synovial Fibroblasts

**Figure 1:**
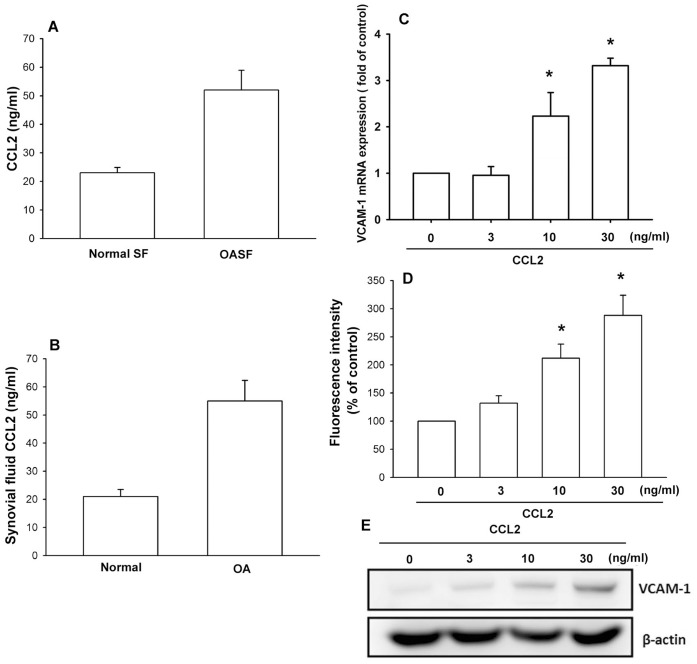
CCL2 increases VCAM-1 expression. (A) Synovial fluid was obtained from normal (n = 12) or osteoarthritis patients (n = 11) and examined with ELISA for the expression of CCL2. (B) Human synovial fibroblasts were cultured for 48 h, and media were collected to measure CCL2 (n = 6). OASFs were incubated with various concentrations of CCL2 for 24 h. The mRNA (C), cell surface (D), and protein expression (E) of VCAM-1 was examined by qPCR, flow cytometry, and Western blotting (n = 4–6). Results are expressed as the mean ± S.E. *: p<0.05 as compared with basal level. #: p<0.05 as compared with CCL2-treated group.

CCL2 has been shown to play important role in OA pathogenesis [16], [17]. Therefore, we wanted to examine human synovial fibroblast tissues for the expression of the CCL2 by using ELISA. Concentrations of CCL2 in synovial fluid were significantly higher in patients with OA than in controls (Fig. 1A). The medium from OASFs showed significant expression of CCL2, which was higher than that in medium from normal SFs (Fig. 1B). Next, we directly applied CCL2 in OASFs and examined the expression of VCAM-1 (an important regulator that promotes monocytes adhesion to endothelial cells). Treatment of OASFs with CCL2 (3–30 ng/ml) for 24 h induced mRNA and cell surface VCAM-1 expression in a concentration-dependent manner, as shown by qPCR and flow cytometry (Fig. 1C&D). In addition, CCL2 also increased VCAM-1 protein expression dose-dependently (Fig. 1E). These data indicate that CCL2 increases VCAM-1 expression in human OASFs.

### The CCL2/CCR2 Axis Promotes VCAM-1 Expression in Human Synovial Fibroblasts

**Figure 2:**
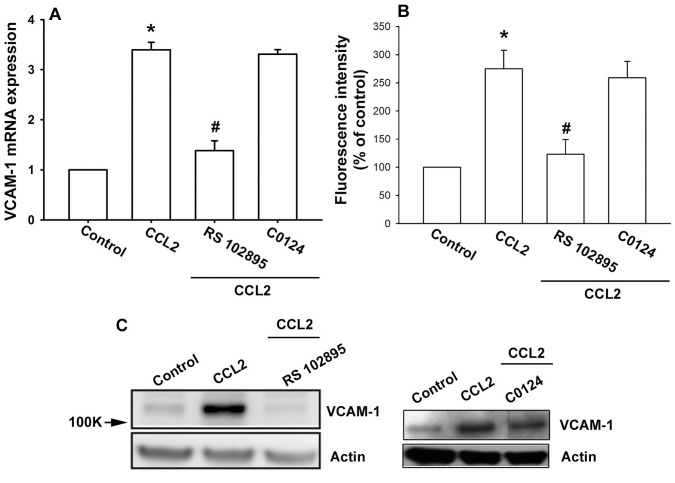
CCL2 increases VCAM-1 expression through CCR2 receptor. OASFs were pretreated for 30 min with RS102895 (400 nM) or C0214 (400 nM) followed by stimulation with CCL2 (30 ng/ml) for 24 h, and VCAM-1 expression was examined by qPCR (A; β-actin was used as internal control), flow cytometry (B), and Western blotting (C) (n = 5–6). Results are expressed as the mean ± S.E. *: p<0.05 as compared with basal level. #: p<0.05 as compared with CCL2-treated group.

Previous studies have shown CCL2 affects cell function through binding to cell surface CCR2 or CCR4 receptor [25], [26]. Pretreatment of cells with CCR2 inhibitor RS102895 but not CCR4 inhibitor C0214 abrogated the CCL2-induced mRNA and cell surface VCAM-1 expression (Fig. 2A&B). In addition, RS102895 but not C0124 blocked the CCL2-increased protein expression of VCMA-1 (Fig. 2C). These results indicate that CCL2 induced VCAM-1 expression through CCR2 receptor in human OASFs.

### The PKCδ and p38MAPK Signaling Pathways are Involved in CCL2-mediated Increase of VCAM-1 Expression

**Figure 3:**
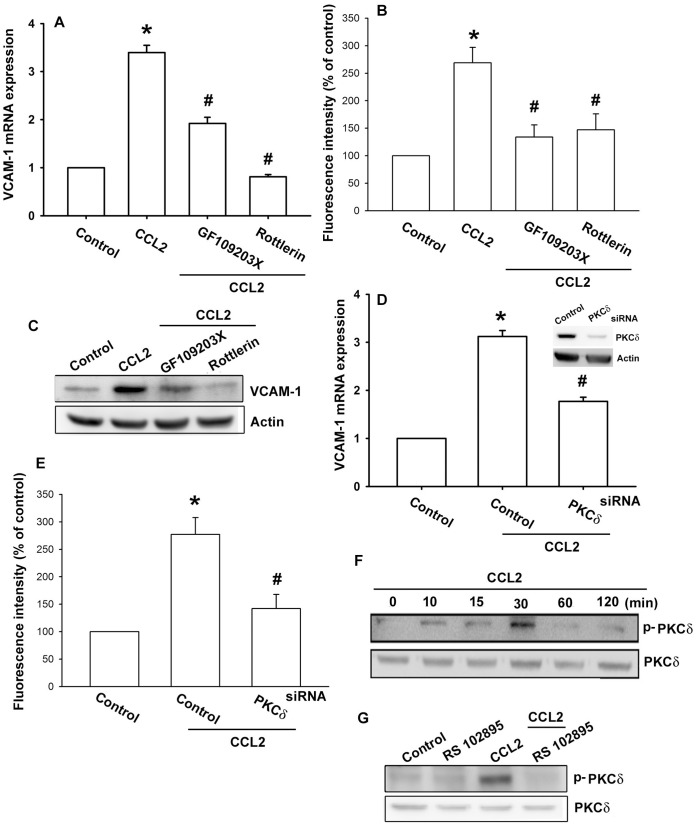
PKCδ is involved in CCL2-induced VCAM-1 expression in synovial fibroblasts. OASFs were pretreated for 30 min with GF109203X (3 µM) or rottlerin (10 µM) followed by stimulation with CCL2 (30 ng/ml) for 24 h, and VCAM-1 expression was examined by qPCR (A), flow cytometry (B), and Western blotting (C) (n = 4–6). OASFs were transfected with PKCδ siRNA for 24 h followed by stimulation with CCL2 for 24 h, and VCAM-1 expression was examined by qPCR (D) and flow cytometry (E). OASFs were incubated with CCL2 for indicated time intervals (n = 4) (F) or pretreated with RS102895 for 30 min before incubation with CCL2 for 30 min (n = 4) (G), and PKCδ phosphorylation was determined by Western blotting (n = 4). Results are expressed as the mean ± S.E. *: p<0.05 as compared with basal level. #: p<0.05 as compared with CCL2-treated group.

PKC has been shown to play an important role in the cellular functions modulated by several stimuli [27], [28]. To determine whether PKC isoforms were involved in CCL2 triggered VCAM-1 expression, OASFs were pretreated with either GF109203X, a pan-PKC inhibitor, or rottlerin, a selective PKCδ inhibitor [29] for 30 min and then incubated with CCL2 for 24 h. As shown in Figure 3A–C, pretreatment with GF109203X and rottlerin reduced CCL2-induced VCAM-1 expression, suggesting that PKCδ may play a role in CCL2-induced VCAM-1 production in OASFs. Transfection of cells with PKCδ siRNA also reduced CCL2-induced VCAM-1 expression (Fig. 3D&E). We then directly measured PKCδ phosphorylation in response to CCL2 and found that stimulation of OASFs led to a significant increase in phosphorylation of PKCδ (Fig. 3F). Pretreatment of cells with RS102895 blocked the CCL2-induced PKCδ phosphorylation (Fig. 3G). Taken together, these results indicate that the CCR2 and PKCδ-dependent pathway is involved in CCL2-induced VCAM-1 expression.

**Figure 4:**
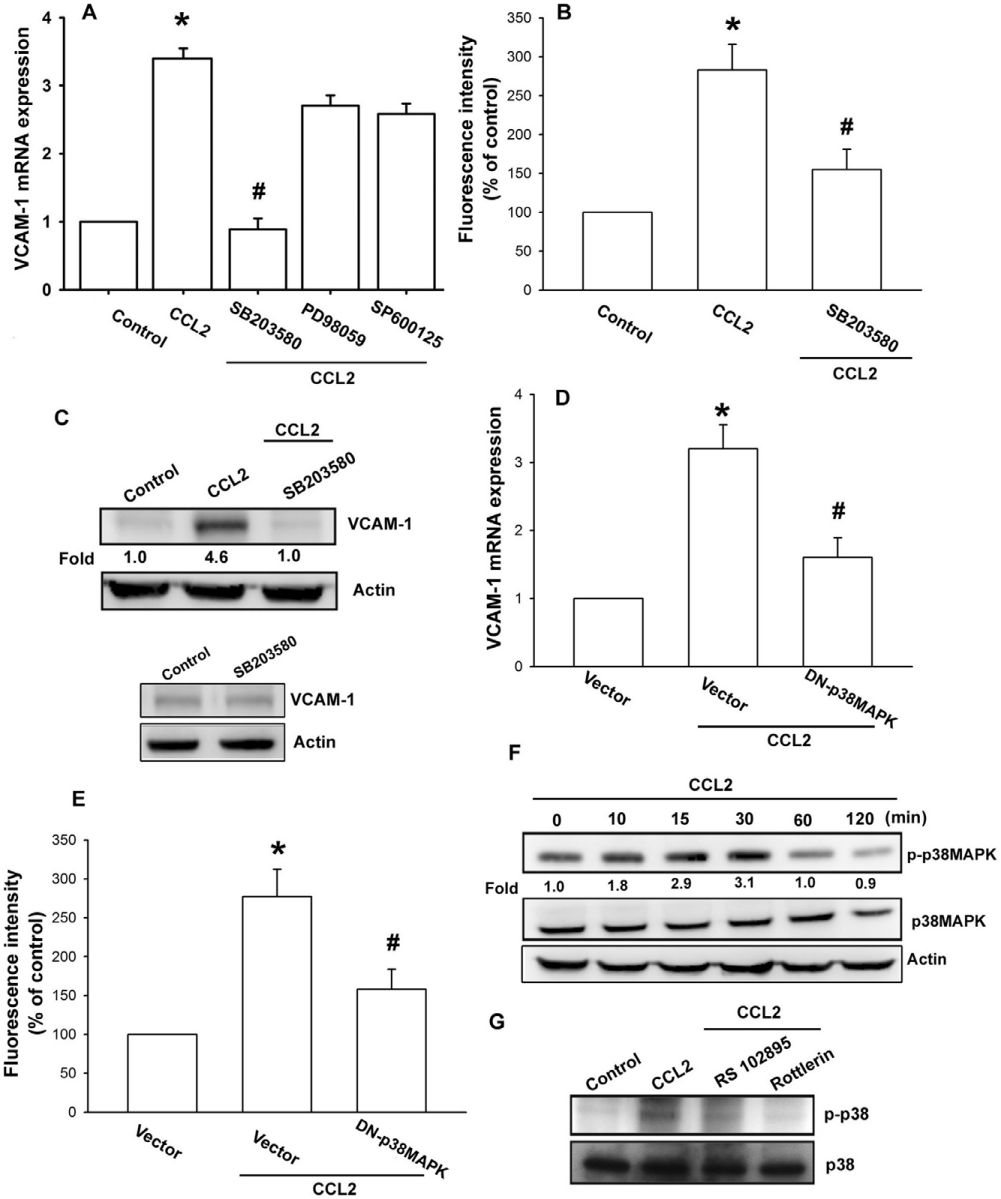
p38MAPK is involved in CCL2-induced VCAM-1 expression in synovial fibroblasts. (A) OASFs were pretreated for 30 min with SB203580 (10 µM), PD98059 (30 µM), or SP600125 (10 µM) followed by stimulation with CCL2 (30 ng/ml) for 24 h, and VCAM-1 expression was examined by qPCR (n = 5). OASFs were pretreated for 30 min with SB203580 followed by stimulation with CCL2 for 24 h, and VCAM-1 expression was examined by flow cytometry (B) and Western blotting (C) (n = 5). OASFs were transfected with p38MAPK mutant for 24 h followed by stimulation with CCL2 for 24 h, and VCAM-1 expression was examined by qPCR (D) and flow cytometry (E) (n = 4). OASFs were incubated with CCL2 for indicated time intervals (F) or pretreated with RS102895 or rottlerin for 30 min before incubation with CCL2 for 30 min (G), and p38 phosphorylation was determined by Western blotting (n = 5). Results are expressed as the mean ± S.E. *: p<0.05 as compared with basal level. #: p<0.05 as compared with CCL2-treated group.

PKCδ-dependent p38MAPK activation is involved in the regulation of VCAM-1 expression [30]. Therefore, we wanted to examine whether CCL2 stimulation enhanced p38MAPK activation in human OASFs. Pretreatment of cells for 30 min with p38 inhibitor SB203580 reduced CCL2-induced VCAM-1 expression (Fig. 4A–C). On the other hand, SB203580 did not affect the basal level of VCAM-1 expression (Fig. 4C; lower panel). In addition, transfection of cells with dominant-negative mutant of p38MAPK also reduced CCL2-mediated VCAM-1 up-regulation (Fig. 4D&E). Furthermore, stimulation of OASFs with CCL2 induced the phosphorylation of p38 in a time-dependent manner (Fig. 4F). Pretreatment of cells with RS102895 and rottlerin blocked the CCL2-induced p38MAPK phosphorylation (Fig. 4G). It has been reported that p38MAPK, ERK, and JNK mediate CCL2 signaling [31], [32]. However, pretreatment of OASFs with ERK inhibitor PD98059 and JNK inhibitor SP600125 only slightly reduced CCL2-increased VCAM-1 mRNA expression (Fig. 4A). Therefore, p38MAPK may be more important than ERK and JNK in CCL2-mediated VCAM-1 expression. Based on these results, it appears that CCL2 acts through a signaling pathway involving the CCR2, PKCδ, and p38MAPK to enhance VCAM-1 expression in human OASFs.

### AP-1 is Involved in the CCL2-mediated Increase of VCAM-1 Expression

**Figure 5:**
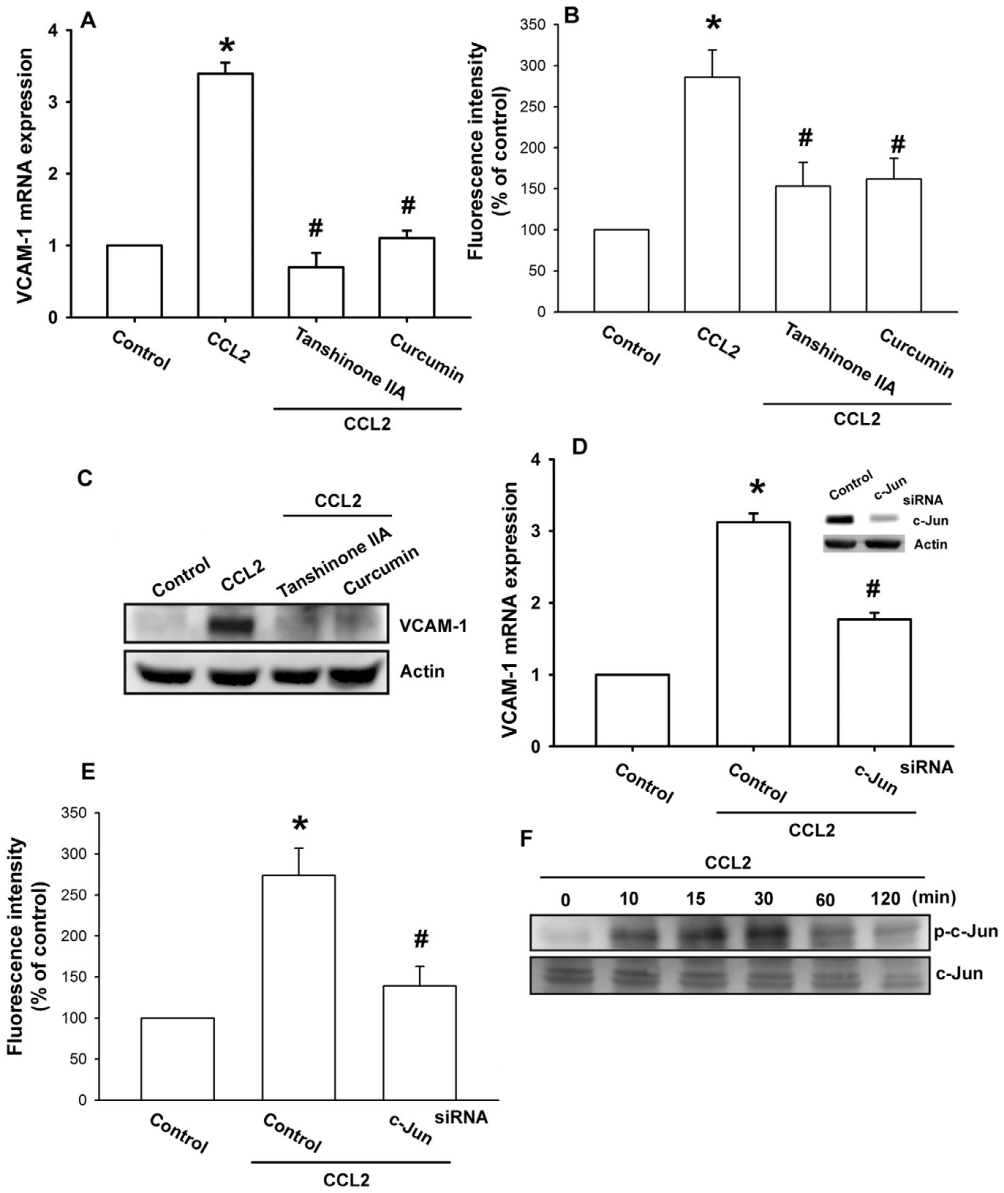
AP-1 is involved in the potentiation of VCAM-1 expression by CCL2. OASFs were pretreated for 30 min with curcumin (3 µM) or tanshinone IIA (5 µM) followed by stimulation with CCL2 (30 ng/ml) for 24 h, and VCAM-1 expression was examined by qPCR (A), flow cytometry (B), and Western blotting (C) (n = 4–6). OASFs were transfected with c-Jun siRNA for 24 h followed by stimulation with CCL2 for 24 h, and VCAM-1 expression was examined by qPCR (D) and flow cytometry (E) (n = 4). (F) OASFs were incubated with CCL2 for indicated time intervals and c-Jun phosphorylation was determined by Western blotting (n = 5). Results are expressed as the mean ± S.E. *: p<0.05 as compared with basal level. #: p<0.05 as compared with CCL2-treated group.

**Figure 6:**
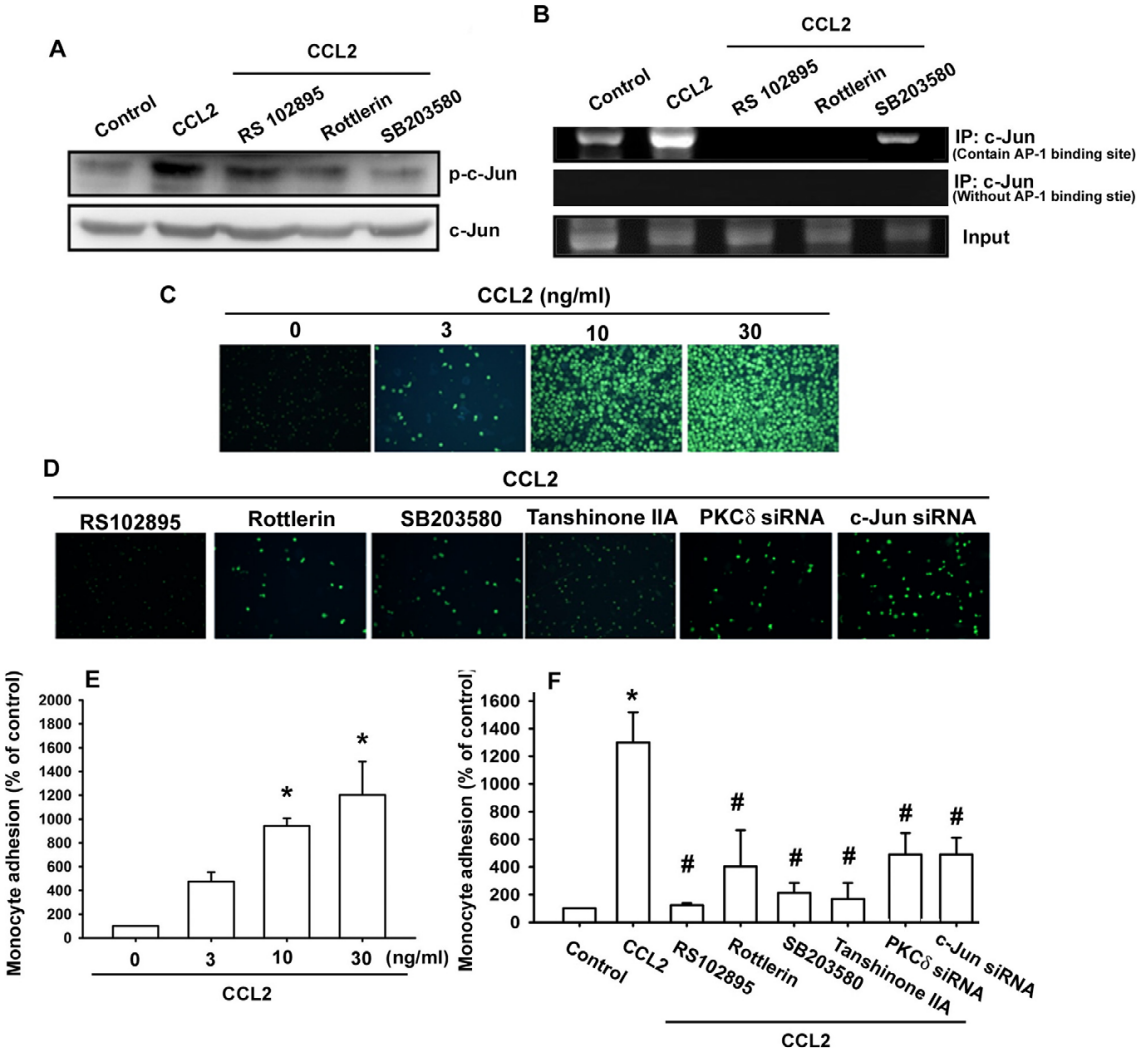
CCL2 induces AP-1 activation and monocytes adhesion through CCR2, PKCδ, and p38 pathway. (A) OASFs were pretreated with RS102895, rottlerin, or SB203580 for 30 min then stimulated with CCL2 for 30 min, and p-c-Jun expression was determined by Western blotting (n = 5). (B) OASFs were pretreated with RS102895, rottlerin, or SB203580 for 30 min then stimulated with CCL2 for 120 min, the chromatin immunoprecipitation assay was then performed (n = 5). OASFs were incubated with various concentrations of CCL2 for 24 h (C&E) or pretreated with RS102895, rottlerin, SB203580, and tanshinone IIA for 30 min or transfected with PKCδ and c-Jun siRNA followed by stimulation with CCL2 for 24 h (D&F) (n = 6). THP-1 cells labeled with BCECF-AM were added to OASFs for 6 h, and then the THP-1 cells adherence was measured by fluorescence microscopy. Results are expressed as the mean ± S.E. *: p<0.05 as compared with basal level. #: p<0.05 as compared with CCL2-treated group.

AP-1 is a transcription factor that plays a crucial role in immune and inflammatory responses. It have been reported that the VCAM-1 promoter includes binding sites for AP-1 [33]. Therefore, we examined the effect of CCL2 on AP-1 transcriptional activation. Pretreatment of cells for 30 min with AP-1 inhibitors (curcumin and tanshinone IIA) inhibited CCL2-induced VCAM-1 expression (Fig. 5A–C). AP-1 activation was further evaluated by analyzing the c-Jun phosphorylation as well as by a chromatin immunoprecipitation assay. Transfection of OASFs with c-Jun siRNA reduced CCL2-mediated increase of VCAM-1 expression (Fig. 5D&E). Stimulation of cells with CCL2 increased c-Jun phosphorylation (Fig. 5F). Pretreatment of cells with RS102895, rottlerin, and SB203580 reduced the CCL2-induced c-Jun phosphorylation (Fig. 6A).

The *in vivo* recruitment of c-Jun to the VCAM-1 promoter (−403 to −30) was assessed via chromatin immunoprecipitation assay [24]. *In vivo* binding of c-Jun to the AP-1 element of the VCAM-1 promoter occurred after CCL2 stimulation (Fig. 6B). The binding of c-Jun to the AP-1 element by CCL2 was attenuated by RS102895, rottlerin, and SB203580 (Fig. 6B). On the other hand, CCL2 stimulation did not increase the binding activity of c-Jun to the VCAM-1 promoter without AP-1 binding site (Fig. 6B). These results indicate that CCL2-induced VCAM-1 expression was mediated through the CCR2, PKCδ, p38MAPK, and AP-1 pathway in human OASFs.

### CCL2 Promotes Monocytes Adhesion through the CCR2, PKCδ, p38MAPK, and AP-1 Pathway

Next, we wanted to measure the monocytes adhesion to OASFs after treatment with CCL2. The adhesion assay was carried out using THP-1 as a monocyte model. Treatment of OASFs with CCL2 enhanced the adhesion between OASFs and THP-1 cells dose-dependently (Fig. 6C&E). In order to determine whether CCR2, PKCδ, p38MAPK, and AP-1 pathway can induce monocytes to adhere to OASFs monolayer, we pretreated of OASFs with RS102895, rottlerin, SB203580, and tanshinone IIA or transfected them with PKCδ and c-Jun siRNA. Both the pretreatment and transfection significantly inhibited the amount of monocytes adhesion (Fig. 6D&F). On the basis of these results, it appears that CCL2 promoted adhesion of monocytes to OASFs through CCR2, PKCδ, p38MAPK, and AP-1 pathway.

## Discussion

OA is a heterogeneous group of conditions associated with defective integrity of articular cartilage as well as related changes in the underlying bone. The chronic inflammatory process is mediated through a complex cytokine network. The factors responsible for initiating the degradation and loss of articular tissues are not completely understood. Although the pathogenesis of the disease remains elusive, up-regulation of adhesion molecules on the surface of the synovial lining may play a key role in recruitment and infiltration of monocytes sites of inflammation in OA [34]. Here we further characterized VCAM-1 as a target protein for the CCL2 signaling pathway that regulates the cell adhesion. We also showed that potentiation of VCAM-1 by CCL2 requires activation of the CCR2, PKCδ, p38MAPK, and AP-1 signaling pathway and promotes monocytes adhesion to OASFs.

The CC-chemokine is regulated on activation of normal T-cell expression, and secreted CCL2 mediates its biological activities through activation of G protein–coupled receptors, CCR2 or CCR4 [25], [26]. It have been reported that CCL2 affects cell migration through binding to cell surface CCR2 or CCR4 receptor [25], [26]. In this study, we found that pretreatment of cells with CCR2 inhibitor but not CCR4 inhibitor blocked CCL2-increased VCAM-1 expression. In addition, CCR2 inhibitor also reduced CCL2-induced monocytes adhesion. The results indicated that expression of CCL2/CCR2 axis was associated with VCAM-1 expression and cell adhesion in OASFs.

Several isoforms of PKC have been characterized at the molecular level and have been found to mediate the progress of OA [35]. We demonstrated that the PKC inhibitor GF109203X antagonized the CCL2-mediated potentiation of VCAM-1 expression, suggesting that PKC activation is an obligatory event in CCL2-induced VCAM-1 expression in these cells. In addition, rottlerin also inhibited CCL2-induced VCAM-1 expression. However, previous report indicated that rottlerin is not a specific PKCδ inhibitor but inhibits may other targets [36]. Therefore, we used PKCδ siRNA to confirm PKCδ function in OASFs. We found that PKCδ siRNA inhibited the enhancement of VCAM-1 expression. Incubation of synovial fibroblasts with CCL2 also increased PKCδ phosphorylation. On the other hand, RS102895 blocked the CCL2-induced PKCδ phosphorylation. These data suggest that the CCR2 receptor and PKCδ pathways are required for CCL2-induced VCAM-1 expression.

p38MAPK has been shown to play an important role in VCAM-1 expression in human synovial fibroblasts [37]. In this study, we used a specific p38MAPK inhibitor SB203580 (10 µM) to examine the role of p38MAPK in CCL2-mediated VCAM-1 expression. SB203580 inhibited p38MAPK at a very low dosage (600 nM) but did not affect ERK or JNK activity at a very high dosage (100 µM). Although there is scant evidence that SB303580 blocks other signaling molecules, we still can not rule out the off-target effect of this chemical inhibitor. In this study, we also used a p38MAPK mutant to confirm the role of p38MAPK in CCL2-mediated VCAM-1 expression. However, siRNA can provide a more specific effect in blocking p38MAPK activation.

There are several binding sites for a number of transcription factors including NF-κB, Sp-1, and AP-1 in the 5′ region of the VCAM-1 gene [38]. Recent studies of the VCAM-1 promoter have demonstrated that VCAM-1 induction by several transcription factors occurs in a highly stimulus-specific or cell-specific manner [30], [39]. The results of our current study show that AP-1 activation contributes to CCL2-induced VCAM-1 expression in synovial fibroblasts. Pretreatment of cells with an AP-1 inhibitors curcumin or tanshinone IIA reduced CCL2-increased VCAM-1 expression. Therefore, the AP-1 binding site is likely to be the most important site for CCL2-induced VCAM-1 production. The AP-1 sequence binds to members of the Jun and Fos families of transcription factors. These nuclear proteins interact with the AP-1 site as Jun homodimers or Jun-Fos heterodimers formed by protein dimerization through their leucine zipper motifs [40]. The results of our study show that CCL2 induced c-Jun phosphorylation. In addition, c-Jun siRNA abolished CCL2-induced VCAM-1 expression in OASFs. Therefore, c-Jun activation mediates CCL2-increased VCAM-1 expression. Furthermore, CCL2 increased the binding of c-Jun to the AP-1 element within the VCAM-1 promoter, as shown by a chromatin immunoprecipitation assay. Binding of c-Jun to the AP-1 element was attenuated by RS102895, rottlerin, and SB203580. These results indicate that the CCL2 may act through the CCR2, PKCδ, p38MAPK, and AP-1 pathway to induce VCAM-1 production in human OASFs.

In conclusion, we have explored the signaling pathway involved in CCL2 induced VCAM-1 expression in human synovial fibroblasts. CCL2 increases VCAM-1 production by binding to CCR2 receptor, and activating PKCδ and p38 which in turn enhances binding of AP-1, resulting in the transactivation of VCAM-1 expression. The CCL2-mediated CCR2, PKCδ, p38MAPK, and AP-1 pathway promotes monocytes adhesion to human OASFs. These findings may provide a better understanding of the mechanisms of OA pathogenesis.
